# Deep Learning for Strain Field Customization in Bioreactor with Dielectric Elastomer Actuator Array

**DOI:** 10.34133/cbsystems.0155

**Published:** 2024-08-14

**Authors:** Jue Wang, Dhirodaatto Sarkar, Atulya Mohan, Mina Lee, Zeyu Ma, Alex Chortos

**Affiliations:** ^1^ School of Mechanical Engineering, College of Engineering, Purdue University, West Lafayette, IN, USA.; ^2^ School of Mechanical Engineering, Key Laboratory of Education Ministry for Modern Design and Rotor-Bearing System, Institute of Design Science and Basic Components, Xi’an Jiaotong University, Xi’an, P. R. China.

## Abstract

In the field of biomechanics, customizing complex strain fields according to specific requirements poses an important challenge for bioreactor technology, primarily due to the intricate coupling and nonlinear actuation of actuator arrays, which complicates the precise control of strain fields. This paper introduces a bioreactor designed with a 9 × 9 array of independently controllable dielectric elastomer actuators (DEAs), addressing this challenge. We employ image regression-based machine learning for both replicating target strain fields through inverse control and rapidly predicting feasible strain fields generated by the bioreactor in response to control inputs via forward control. To generate training data, a finite element analysis (FEA) simulation model was developed. In the FEA, the device was prestretched, followed by the random assignment of voltages to each pixel, yielding 10,000 distinct output strain field images for the training set. For inverse control, a multilayer perceptron (MLP) is utilized to predict control inputs from images, whereas, for forward control, MLP maps control inputs to low-resolution images, which are then upscaled to high-resolution outputs through a super-resolution generative adversarial network (SRGAN). Demonstrations include inputting biomechanically significant strain fields, where the method successfully replicated the intended fields. Additionally, by using various tumor–stroma interfaces as inputs, the bioreactor demonstrated its ability to customize strain fields accordingly, showcasing its potential as an advanced testbed for tumor biomechanics research.

## Introduction

Cell behavior is governed by a combination of internal and external factors, including both chemical and mechanical signals. The design and implementation of bioreactors, particularly cell stretchers, are pivotal in applying physical biomechanical stimuli—stretching, compression, or torsion—to cells or tissues [1]. Such devices are adept at replicating the mechanical environments experienced during physiological activities like heartbeats, vascular pulsation, and respiratory movements. Understanding the complex mechanical stimuli is essential for unraveling mechanobiology’s role in maintaining healthy tissues and investigating diseases like cardiac fibrosis [2–5], idiopathic pulmonary fibrosis [6,7], musculoskeletal disorders [8], and connective tissue diseases [9]. Moreover, these devices facilitate studies on how cells alter their morphology, structure, and function under varying mechanical stimuli and how these transformations impact cell growth, differentiation, and apoptosis [10].

Current bioreactors are typically powered by pneumatic actuators [11–13], motors [14–16], dielectric elastomer actuators (DEAs) [17,18], or shape memory alloys (SMAs) [19], with a common limitation being the scarcity of independently drivable actuators. This constraint restricts them to generating simple uniaxial [20,21], equibiaxial [22–25], and non-equibiaxial [11] stretching. However, the demand in fields such as cardiovascular biomechanics [26], tissue engineering and regenerative medicine [27], and tumor biomechanics [28] necessitates bioreactors capable of producing more complex and customized strain fields. These advanced strain fields serve as testbeds for in vitro cellular experiments, requiring a bioreactor with an actuator array where each actuator can be independently controlled. Given the size constraints of pneumatic actuators and motors for centimeter-scale bioreactors and the slow response time of SMAs, DEA arrays emerge as the optimal solution for achieving the desired complexity and customization of strain fields.

With the advent of smart materials and cutting-edge fabrication technologies, the creation of DEA arrays has become feasible. Hajiesmaili and Clarke [29] achieved an optically addressable 6 × 6 DEA array using embedded percolative networks of zinc oxide nanowires with each actuator independently controlled. The DEA arrays have been applied to tactile display [30] and single-cell mechanotransduction to generate uniaxial strain [31]. However, controlling these DEA arrays to achieve customized strain fields presents a significant challenge. The nonlinear characteristics of DEAs, coupled with the inter-actuator interactions, are complex to encapsulate within an analytical model. In recent years, the emergence of machine learning technology has revolutionized control and sensing within the soft robotics domain [32,33]. This evolution has introduced sophisticated methods for enhancing robotic responsiveness and adaptability. Subsequently, within the subset of soft robotics, the shape morphing devices, the relationship between the inputs to an array of actuators, and their 2.5-dimensional (2.5D) or 3D deformations can be delineated through 1D vector regression [34,35] or 3D point cloud regression [36]. Inspired by these advancements, for 2D deformations in DEA arrays, the mapping between strain field images and input arrays can be effectively established through image regression. Yang et al. [37] proposed the utilization of generative adversarial networks (GANs) for the direct prediction of stress and strain fields. Park et al. [38] utilized the multiscale kernel neural network to achieve similar functionality. However, for the application described previously, predicting control inputs based on stress and strain fields plays a more important role.

In this study, we designed a bioreactor equipped with a 9 × 9 array of independently controllable DEAs. Our goal was to predict the strain fields generated by this bioreactor or replicate given target strain fields using a machine learning-based image regression approach. Initially, we collected data through the establishment of a finite element analysis (FEA) simulation model. In the FEA, the device was prestretched, followed by the random assignment of voltages to each pixel, yielding 10,000 distinct output strain field images for the training set. In the training phase, we employed a multilayer perceptron (MLP) to achieve inverse control, enabling the device to replicate a specified target strain field based on input images. Furthermore, we combined MLP with super-resolution generative adversarial network (SRGAN) to facilitate rapid prediction of strain field images from input voltage arrays. In the demonstration section, we input 2 biomechanically significant strain fields, and the proposed method successfully enabled the virtual device to replicate these fields. Subsequently, introducing various tumor–stroma interfaces as inputs, the virtual device adeptly replicated these strain fields as well, demonstrating its capacity to customize strain fields based on different tumor–stroma interfaces.

The key novel contributions are summarized as follows:

• This paper innovatively proposes the use of an image regression method to achieve control over the strain field, enabling the replication of strain fields based on input images.

• The forward control implemented in this paper can rapidly and accurately predict strain field images based on control input.

• The inverse control implemented in this paper enables the customization of strain fields according to the practical requirements of biomechanics applications, particularly by replicating identical strain fields based on tumor–stroma interfaces.

## Materials and Methods

### Design of the bioreactor with DEA array

**Fig. 1.**
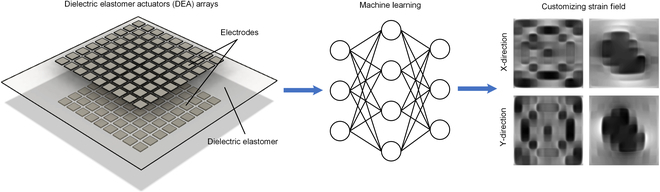
The bioreactor with DEA array can achieve strain field prediction and customization by using image regression-based machine learning.

In this study, we introduce the design of a bioreactor capable of generating complex strain fields for the sophisticated stretching of cells or tissues. This system features a 9 × 9 array of independently controllable DEAs spread over a 100 mm × 100 mm dielectric elastomer membrane. The 9 × 9 array is large enough to demonstrate the value of machine learning (it would be challenging to find an analytical solution) and is sufficient for the envisioned application in bioreactors. The varied actuation of individual DEAs, combined with intricate coupling between actuators, enables the creation of complex strain fields. As illustrated in Fig. 1, the primary objective of this work is to develop an image regression-based machine learning model to predict and customize complex strain fields.

**Fig. 2.**
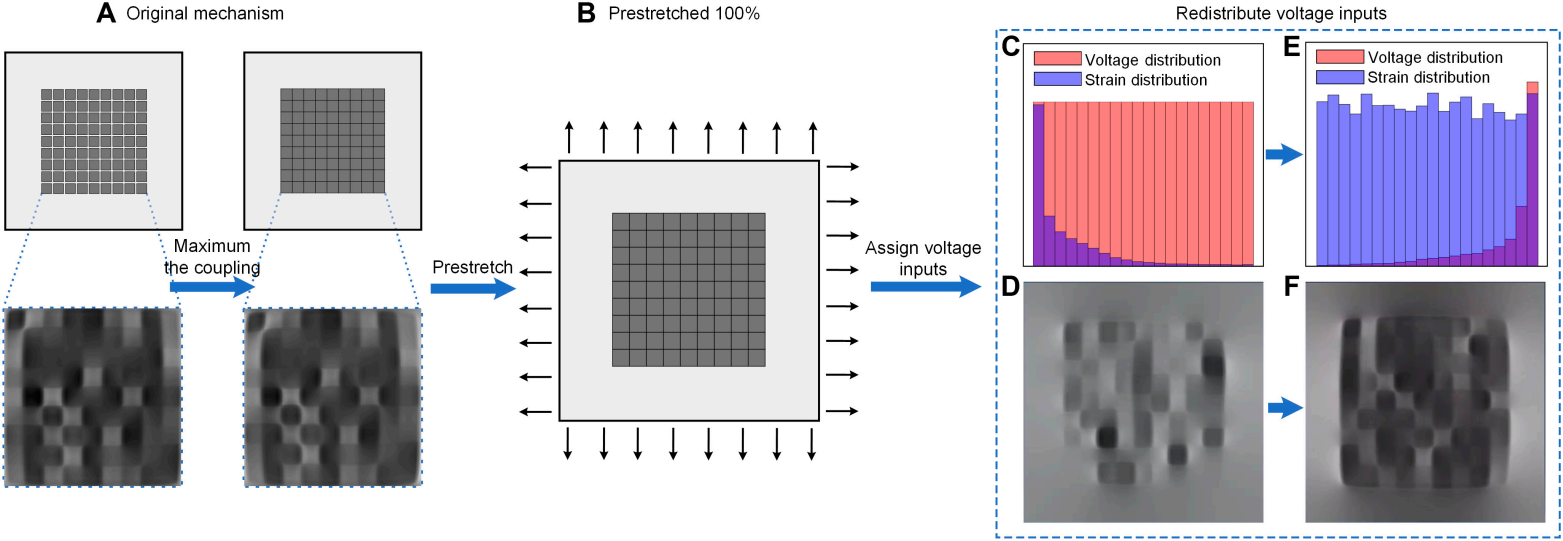
The FEA simulation of the proposed bioreactor for training data collection. (A) Original mechanism with no gap between pixels to maximize the actuation coupling between actuators. (B) The device is prestretched for 100% before assigning the voltage to the pixels. (C to F) Re-distributing the voltage inputs to make sure the strain distribution in the simulation result can be uniform, where (C) is the voltage and strain distribution before the voltage redistribution, (D) is one of the simulation results with nonuniform strain distribution, (E) is the voltage and strain distribution after the voltage redistribution, and (F) is one of the simulation results with uniform strain distribution.

In physical devices, gaps between pixels will be necessary for electrical isolation. However, the core of our work focuses on customizing the strain field using machine learning-based methods. The essence of these machine learning approaches is to decipher the mutual coupling between pixels during actuation. Greater coupling presents significant challenges for analytical modeling, making machine learning approaches particularly valuable. By maximizing this coupling, we not only underscore the significance of using machine learning but also ensure that our method can handle cases with less coupling more effectively. As shown in Fig. 2A, smaller gaps allow the deformation of one pixel to more significantly affect its neighbors, thereby maximizing coupling and achieving the objectives mentioned earlier.

### Data collection from simulation results

Data collection from a physical device might appear as the most direct approach. Yet, the repeatability of actuator arrays is limited, as they are prone to accumulate errors after thousands of actuations. Moreover, inherent fabrication and systematic errors of the physical device can also diminish training precision. An alternative data collection method involves constructing an accurate FEA model for the device and harvesting training data from simulation results. This technique not only yields a substantial volume of training data swiftly but also ensures greater data stability, resulting in a more precise model.

Here, we utilize Abaqus to model our DEA array. The simulation process is divided into 2 steps. The initial step involves prestretching the DEAs, which significantly enhances the actuators’ strain capability. This prestretch is necessary for the devices to remain planar during actuation since the actuation in each pixel causes in-plane expansion. Without prestretch, the devices would exhibit complex deformations such as out-of-plane buckling. We configure the first step with the “general, static” preset, applying a displacement in both the X and Y directions. This displacement equals the original length of the devices in the X and Y directions. This step provides a 100% engineering strain on both directions, and the true stain is 69.31%. The results of this first step are then used as the starting point for the subsequent step in the simulation process. In the real devices, the prestretch will be done in the fabrication process before the membrane is mounted to the base. The cells will be cultured on the membrane after the membrane has been prestretched, so the prestretch strain will not be counted in the strain applied to the cells (Fig. 2A and B).

In the second step of the simulation, we employ a custom user element (UEL) subroutine that uses temperature as a proxy for voltage for DEA simulation [39]. The subroutine calculates the material’s strain and electric field (input as temperature) based on nodal displacements and electric potential. It then computes the Maxwell stress, reflecting the contribution of the electric field to the stress and demonstrating the electromechanical coupling effect. These calculated values of strain and Maxwell stress are used to update the residual vectors and the stiffness matrix, effectively simulating the electromechanical behavior of the DEA material.

The UEL subroutine introduces the effect of voltage input on mechanical deformations by incorporating this coupling into the stiffness matrix and residual vectors of the finite element formulation. Implementing this in Abaqus requires a step where mechanical deformation depends on another parameter. Since we define the element’s behavior, the parameter’s label in the software is irrelevant; it merely serves as a method for data transfer. The coupled temperature–displacement step in Abaqus allows this process, where “temperature” acts as a variable that the UEL treats as voltage, yielding the desired outputs.

Since the simulation does not account for the material’s viscoelastic properties, it represents the quasi-static deformation of the DEA rather than a dynamic process. The material we choose for DEA is Ecoflex. In Abaqus setting-ups, the material stress–strain model is set to neo-Hookean model with *C*_1_0 = 0.207, *D*_1_ = 0.05 [40]. Since our final strain, including prestretch and DEA actuation, is at most around 95%, we do not need more complex and higher-order hyperelastic models such as Mooney–Rivlin or Ogden 3rd order. These models show minimal differences compared to the neo-Hookean model within 100% strain, and the complex models would also increase computation time. While the time saved for a single simulation may be limited, we need to generate 10,000 simulation datasets for training, making the overall reduction in computation time substantial. The environment is set to “coupled temp-displacement”. We sequentially assign a voltage array as temperature boundary conditions to each pixel’s top electrode, while the bottom electrode is uniformly set to 0 degrees. Subsequently, fixed constraints are applied to the device to keep one endpoint static and both lateral faces parallel to the X–Z and Y–Z planes, respectively, with the bottom face parallel to the X–Y plane. For meshing, we opt for element type C3D8T—an 8-node thermally coupled brick with trilinear displacement and temperature.

In our research, the goal is to gather training data through simulations by ensuring a random strain field in each case. Additionally, we aim for a uniform strain distribution in every case, meaning that it should consistently encompass all strain levels from the lowest to the highest value. A model trained with a training set distributed in this manner can achieve better generalization, meaning that when a new data or image (not part of the training set) is used as input, its output can have higher accuracy. The relationship between voltage and strain for DEA is not linear. We cannot use uniform voltage distribution to get a uniform strain distribution. To address this, we first apply various voltages to a single pixel to establish a nonlinear voltage–strain curve. We then employ a dual Gaussian fit to precisely define the relationship between voltage and strain by using Matlab Curve Fitting Toolbox, as shown in the following:

$$\begin{array}{c} \epsilon =a_{1}\text{exp}\left(-{\left(\frac{V-b_{1}}{c_{1}}\right)}^{2}\right)+a_{2}\text{exp}\left(-{\left(\frac{V-b_{2}}{c_{2}}\right)}^{2}\right)\;, \end{array}$$ (1)

where *ϵ* and *V* refer to the strain and voltage, respectively. The fitting parameters are: *a*_1_ = 7.756*e* + 15; *b*_1_ = 24.1; *c*_1_ = 2.964; *a*_2_ = 47.69; *b*2 = 10.18; *c*2 = 4.401. This fitting achieved within our operational range resulted in an *R*^2^ value of 0.9998.

If we want to achieve a uniformly distributed strain field, the voltage distribution we use cannot be uniform. If we use a uniformly distributed voltage, as shown by the red bar in Fig. 2C, the resulting strain field distribution, indicated by the red and blue bars in Fig. 2C, is very uneven. We then adjust the distribution of voltage inputs to comply with the aforementioned fitting equation, as shown by the red bar in Fig. 2E. This distribution of voltage inputs results in a very uniform strain field distribution, as shown by the blue bar in Fig. 2E. Figure 2D and F shows the final strain field distribution resulting from the voltage distributions before and after optimization, respectively.

To quantify the importance of this step for the strain field distribution, we generated 10 simulations using the original voltage distribution and 10 simulations using the optimized voltage distribution. From each resulting strain field image, we extracted the strain at the center point of each pixel, creating an 81D strain array for each image. We then calculated the standard deviation of probabilities and entropy of these arrays to quantify the uniformity of the strain distributions.

The results show that with the original voltage distribution (uniform linear distribution), the standard deviation of probabilities for the strain distribution in the device was 0.165, and the entropy was 1.35. With the optimized voltage distribution, the standard deviation of probabilities decreased to 0.015, and the entropy increased to 1.94. The smaller standard deviation indicates a more even distribution, while the higher entropy further suggests a more uniform distribution.

To this end, we randomly generate 10,000 sets of 81D voltage input arrays, each of which is then assigned to the simulation model. This process yields 10,000 corresponding strain field images, which, along with the input voltage arrays, form our training dataset. Additionally, we generate an extra 100 sets of voltage input arrays to serve as a test batch. Once we obtain the simulation results for all training and test sets, we export the images representing LE11 and LE22, which are the X-direction and Y-direction strains, respectively, to form another part of our training and testing dataset. Given our intended application of customizing strain fields—either reproducing them from image inputs or predicting them from voltage inputs—we directly collect these images for our training set and proceed with regression training on them. To facilitate the training of our model, we ensure that the exported strain field images are in grayscale.

### Machine learning-based control

#### Inverse control for customizing strain field

**Fig. 3.**
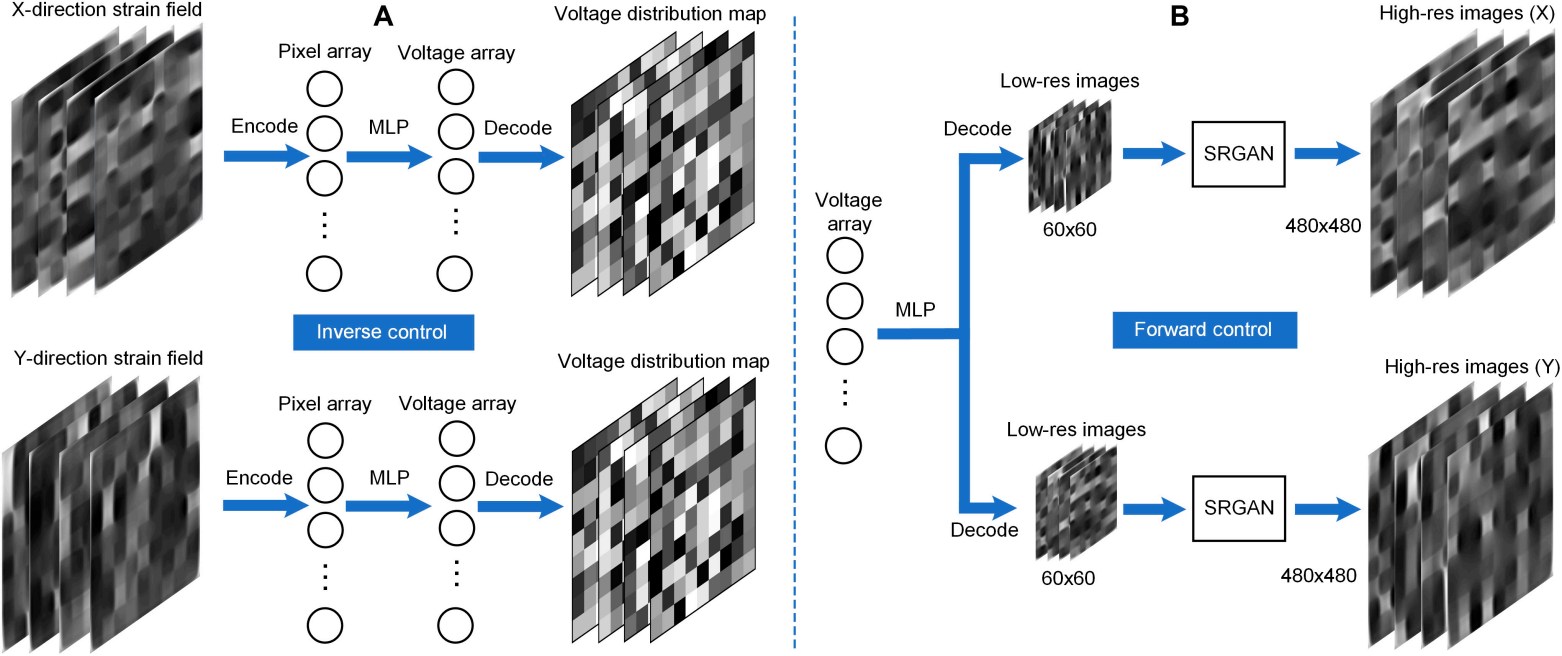
The machine learning architecture for customizing and predicting the strain field in bioreactor with DEA array. (A) Inverse control architecture, which can reproduce the strain field by inputting target strain field images. (B) Forward control architecture, which can predict the strain field by inputting the voltage input array for DEA array.

Prior to initiating the machine learning process, we first preprocess the image data. This involves cropping the images to retain only the central deformed region and then normalizing them. Our initial phase of training focuses on inverse control, which entails using image inputs to predict the voltage inputs that would result in the corresponding X-direction and Y-direction strain distributions. Training is conducted separately for X-direction and Y-direction strains because the voltage inputs required to reproduce target images in X-direction strain fields differ from those needed for Y-direction strain fields. During training, all images are resized to 60 × 60 pixels and then flattened into 3,600D vectors. These vectors are then regressed against the 81D voltage input vector using an MLP. Our regression model incorporates a hidden layer with 180 nodes and utilizes the “tanh” activation function. The optimizer chosen is stochastic gradient descent (SGD), with a learning rate set to 0.001 (Fig. 3A)

#### Forward control for predicting strain field

In this work, “forward control” refers to predicting the strain field that would be formed by a given voltage input array. This process is akin to simulation, but while FEA typically requires an extended period to simulate a strain field—for our case, approximately 2 to 3 min per simulation, which precludes real-time prediction—the use of a pretrained model can achieve image prediction in roughly 0.1 s. This represents a significant reduction in time complexity compared to FEA. Our proposed machine learning architecture for forward control is a 2-stage process. The first stage involves regression from the voltage input array to a low-resolution (LR) image. Here, all training set images are resized to 60 × 60 pixels and flattened into 3,600D vectors, which are then mapped from the 81D input to the 3,600D output via an MLP, with parameter settings similar to those in the inverse control. Here, in the first step, we employ a single model to concurrently predict LR images of both X-direction and Y-direction strains. This is achieved by concatenating the two 3,600D vectors representing *x* and *y* strains into a single 7,200D vector, which serves as the output during training. The second stage involves decoding the 3,600D vector back into a 60 × 60 LR image and using an SRGAN to regress it against the original 480 × 480 high-resolution (HR) images. During the second step of the forward control, as shown in Fig. 3B, it is imperative to train the X and Y data separately due to the significant differences in the pixel distribution patterns of their strain field images. The combined effect of these 2 stages enables the prediction of a 480 × 480 HR strain field image from an 81D voltage input array.

In the SRGAN, the model comprises 2 key components. The first is the generator, which is tasked with creating HR images. It features a succession of residual blocks that learn the mapping from LR to HR, capped with upsampling blocks to enhance the resolution of the input images. The generator’s goal is to fabricate high-quality HR images from LR inputs. The second component is the discriminator, designed to discern between authentic HR images and the synthetic HR images fabricated by the generator. This is achieved through a sequence of convolutional blocks, each succeeded by a LeakyReLU activation to introduce nonlinearity, and batch normalization to ensure stability. Throughout the training phase, we utilize an NVIDIA RTX 3090 graphics card for computation and adopt binary cross-entropy (BCE) as the loss function for GAN training. The optimizer of choice is Adam. To align the training pace of the generator and discriminator, we set the learning rate for the generator at 0.00025 and for the discriminator at 0.0001. We fix the batch size at 8 and run the model for a total of 50 epochs. On average, each epoch takes about 11 min to complete. Optimal training precision is generally achieved around the 20th epoch and maintains consistency thereafter.

In our forward control strategy, we avoid directly regressing the 81D voltage input array against HR images due to the substantial increase in vector dimensions when HR images are flattened. This approach would require significant memory allocation and extend training durations, making it impractical without access to high-performance computing resources.

## Results

### Model performance of inverse control

In the inverse control, we applied test datasets to their 2 respective pretrained models. We achieved a prediction accuracy of 88.79% with a mean squared error (MSE) of 0.189 kV for the X-direction strain, and an accuracy of 87.31% with an MSE of 0.211 kV for the Y-direction strain. Considering our input voltage range of 0 to 7.1 kV, these errors are remarkably low.

**Fig. 4.**
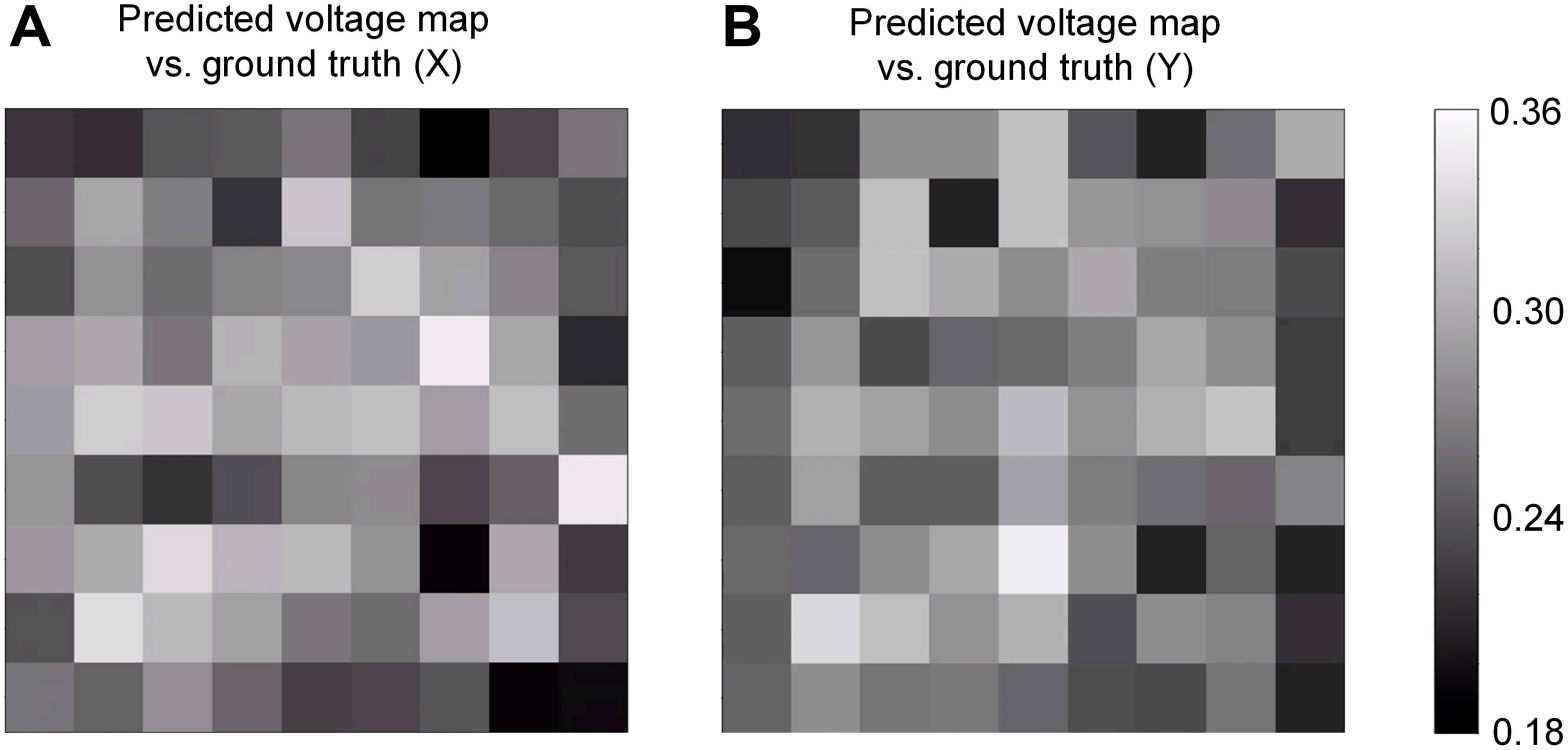
The model performance of the inverse control. (A) Comparing the predicted voltage map with the ground truth for the model of X-direction strain. (B) Comparing the predicted voltage map with the ground truth for the model of Y-direction strain.

To visualize the model performance of the inverse control, we converted the predicted voltage arrays and the ground truths (data collected from simulation) from the 100 test sets into 9 × 9 matrices to compute their corresponding error matrices. These error matrices were then visualized as 9 × 9 grid maps, as illustrated in Fig. 4, where darker squares indicate smaller errors and lighter squares signify larger errors. It was observed that, aside from a few pixels with an average error exceeding 0.3, most errors fell within the range of 0.18 to 0.3. Interestingly, the error performance of pixels at the edges was better compared to other areas. This could be attributed to the edge pixels being less influenced by the coupling effect of an actuator on one side, thereby allowing their characteristics to be learned with greater precision.

### Model performance of forward control

For the evaluation of our forward control’s performance, visualization of the similarity between the generated images and the ground truth is essential. To assess the degree of similarity between images, we employed the structural similarity index measure (SSIM), which aims to provide an image quality assessment that more closely aligns with human visual perception than traditional pixel-based metrics, such as MSE. Unlike MSE, SSIM takes into account factors like image structure, brightness, and contrast, offering a more comprehensive and accurate evaluation of image quality. The principle behind its calculation for 2 images, x and y, is defined as follows [41]:

$$\begin{array}{c} \text{SSIM}\left(x,y\right)=\left(\frac{2\mu_{x}\mu_{y}+C_{1}}{\mu_{x}^{2}+\mu_{y}^{2}+C_{1}}\right)\left(\frac{2\sigma_{x}\sigma_{y}+C_{2}}{\sigma_{x}^{2}+\sigma_{y}^{2}+C_{2}}\right)\left(\frac{\sigma_{\mathrm{xy}}+C_{3}}{\sigma_{x}\sigma_{y}+C_{3}}\right) \end{array}$$ (2)

where *μ_x_* and *μ_y_* are the average luminance of image *x* and *y*, respectively; $\sigma_{x}^{2}$ and $\sigma_{y}^{2}$ are the pixel variance of image *x* and *y*, respectively; *σ_xy_* is the pixel covariance of image *x* and *y*; and *C*_1_, *C*_2_, *C*_3_ are small constants added to avoid division by zero with *C*_3_ typically set to *C*_2_/2. The above formula can be decomposed into 3 parts. The luminance comparison function is as follows: $\frac{2\mu_{x}\mu_{y}+C_{1}}{\mu_{x}^{2}+\mu_{y}^{2}+C_{1}}$, which compares the brightness of the 2 images. The contrast comparison function is as follows: $\frac{2\sigma_{x}\sigma_{y}+C_{2}}{\sigma_{x}^{2}+\sigma_{y}^{2}+C_{2}}$, which compares the contrast of the 2 images. The structure comparison function is as follows: $\frac{\sigma_{\mathrm{xy}}+C_{3}}{\sigma_{x}\sigma_{y}+C_{3}}$, which compares the structural information of the 2 images.

In our computations, we initially set a Gaussian window of size 11. For every local region within the image, convolution with the Gaussian window allows for the calculation of the region’s weighted average luminance, variance, and covariance. The Gaussian window ensures that the contribution of center pixels to the outcome is greater than that of edge pixels, mirroring the characteristics of human visual perception, which tends to focus more on details around the focal point. Within each local window, the SSIM value for the 2 images is calculated based on the derived weighted average luminance, variance, and covariance. The Gaussian window is then slid across the entire image, repeating the aforementioned steps for every possible local area, thereby generating a collection of local SSIM values across the image. Finally, an overall SSIM value for the 2 images is obtained by averaging all the local SSIM values.

**Fig. 5.**
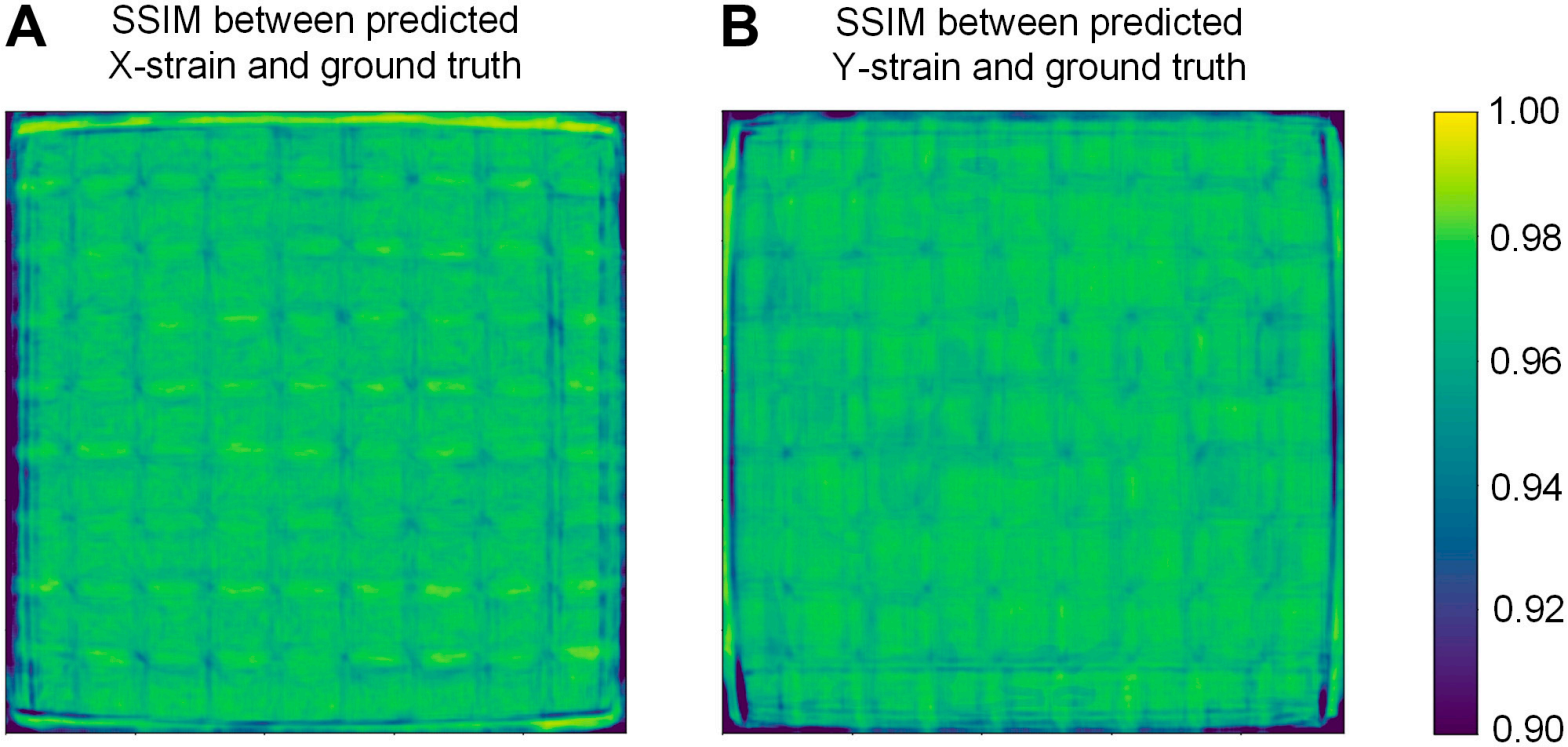
The model performance of the forward control. (A) SSIM between the predicted X-direction strain field image and the ground truth. (B) SSIM between the predicted Y-direction strain field image and the ground truth.

For the prediction of strain images in both the X-direction and Y-direction, the SSIM achieved values of 0.9664 and 0.9673, respectively, indicating a predictive accuracy to a level where differences are barely discernible to the naked eye. Subsequently, we aggregated all the local SSIM values into a matrix, which was visualized in Fig. 5. It was observed that, aside from the nondeformed edge areas where the SSIM was below 0.9, the SSIM values in other regions exceeded 0.95.

### Reproducing complex strain field with image input

**Fig. 6.**
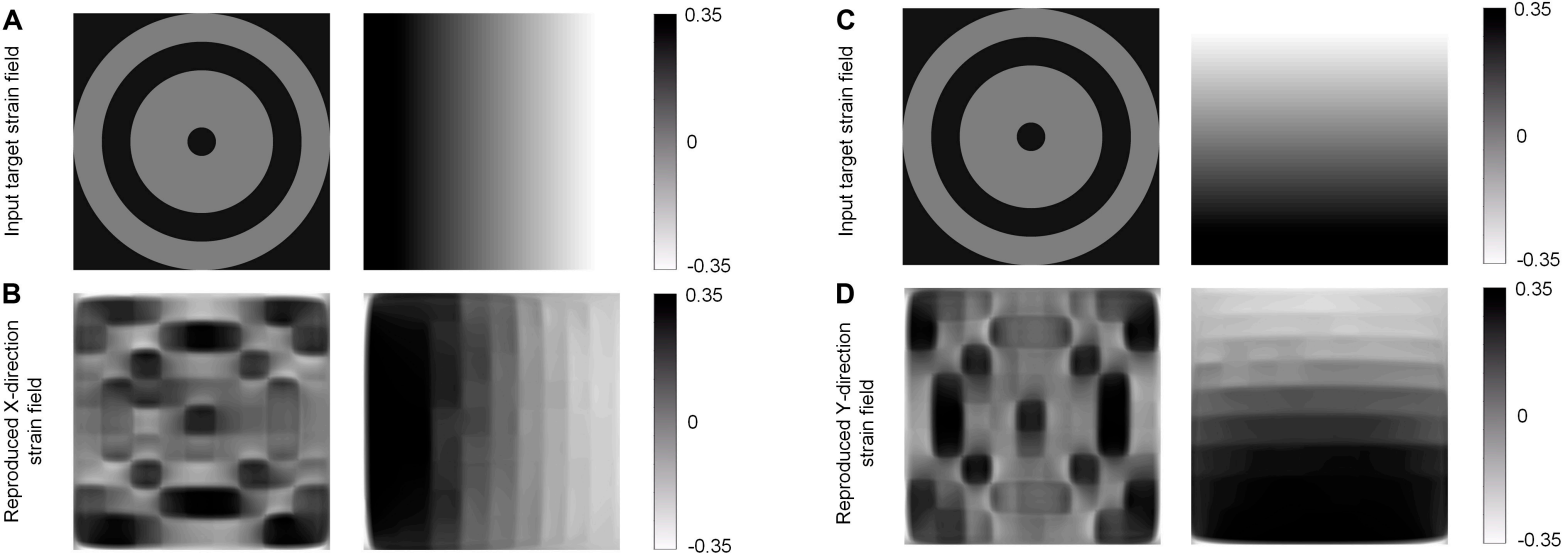
Demonstrations of inverse control to reproduce strain fields with multiple annuli of varying strains and unidirectional gradient variations. (A) Target strain fields with multiple annuli of varying strains and horizontal gradient variations for reproducing X-direction strain field. (B) Reproduced X-direction strain field. (C) Target strain fields with multiple annuli of varying strains and horizontal gradient variations for reproducing Y-direction strain field. (D) Reproduced Y-direction strain field.

Current bioreactors primarily generate uniaxial, equibiaxial, and non-equibiaxial strains, lacking the capability to produce more complex strain fields. However, in practical applications, as illustrated in Fig. 6, strain fields with multiple annuli of varying strains and unidirectional gradient variations hold significant biological relevance. For instance, multi-annular strain fields with varying strains across different rings can simulate the strain distribution in circular tissue structures like blood vessels, intestines, or certain cartilaginous tissues. Similarly, strain fields with unidirectional gradient changes can mimic the strain distribution in muscular tissues, tendons, and some connective tissues, as well as study cell migration under different strain gradients, which is crucial for understanding how cells respond to varying strain environments during wound healing. Yet, contemporary bioreactors are unable to generate such complex strain fields. Thus, we designed 2 target images representing these scenarios and input them into the inverse control pretrained models for the X-direction and Y-direction. The voltage input arrays obtained were then applied to our virtual device, successfully generating the strain fields depicted in Fig. 6. We compared the similarity between the target complex strain field with the reproduced strain field by using SSIM, and the result is shown in Table.

**Table.** The SSIM between target strain field with reproduced strain field

	SSIM between target and X-strain field	SSIM between target and Y-strain field
Circular strain field	0.7500	0.7406
Gradient strain field	0.8335	0.7898
Tumor demo 1	0.8809	0.8915
Tumor demo 2	0.8828	0.8952
Tumor demo 3	0.8682	0.9108
Tumor demo 4	0.9014	0.8903

Comparing Fig. 6B and D with Fig. 6A and C, it is evident that the virtual device, within its resolution constraints, can accurately replicate the target strain fields as proposed by our inverse control method. However, it is important to note that the range of strain fields the device can generate is limited. For instance, in the case of X-direction strain, the device can perfectly replicate strain fields with horizontal gradient distributions, but it struggles to ideally generate strain fields with vertical gradient distributions. Similarly, for Y-direction strain, achieving horizontal distributions poses a challenge.

### Mimicking the shape of tumor–stroma interface for cancer cell testbed

Another meaningful application of the method proposed in this paper is the generation of strain fields corresponding to the 2D shape of tumor–stroma interface. This type of bioreactor holds significant promise for tumor biology and cancer treatment research. Tumor tissues are subject to a unique biomechanical environment that differs significantly from the uniaxial or biaxial stretching typically experienced by heart or lung cells. The mechanical environment of tumors is closely related to their interaction with surrounding organs or biological tissues at the tumor–stroma interface. Simulating the shape of this interface can be beneficial for multiple reasons. This allows the study of how tumor growth and expansion exert mechanical stress on surrounding tissues, impacting the behavior of adjacent tissues and cells. By replicating the strain corresponding to the tumor–stroma interface shape, experiments can be conducted in vitro without the need for actual tumors, providing a highly valuable testbed for research [42].

**Fig. 7.**
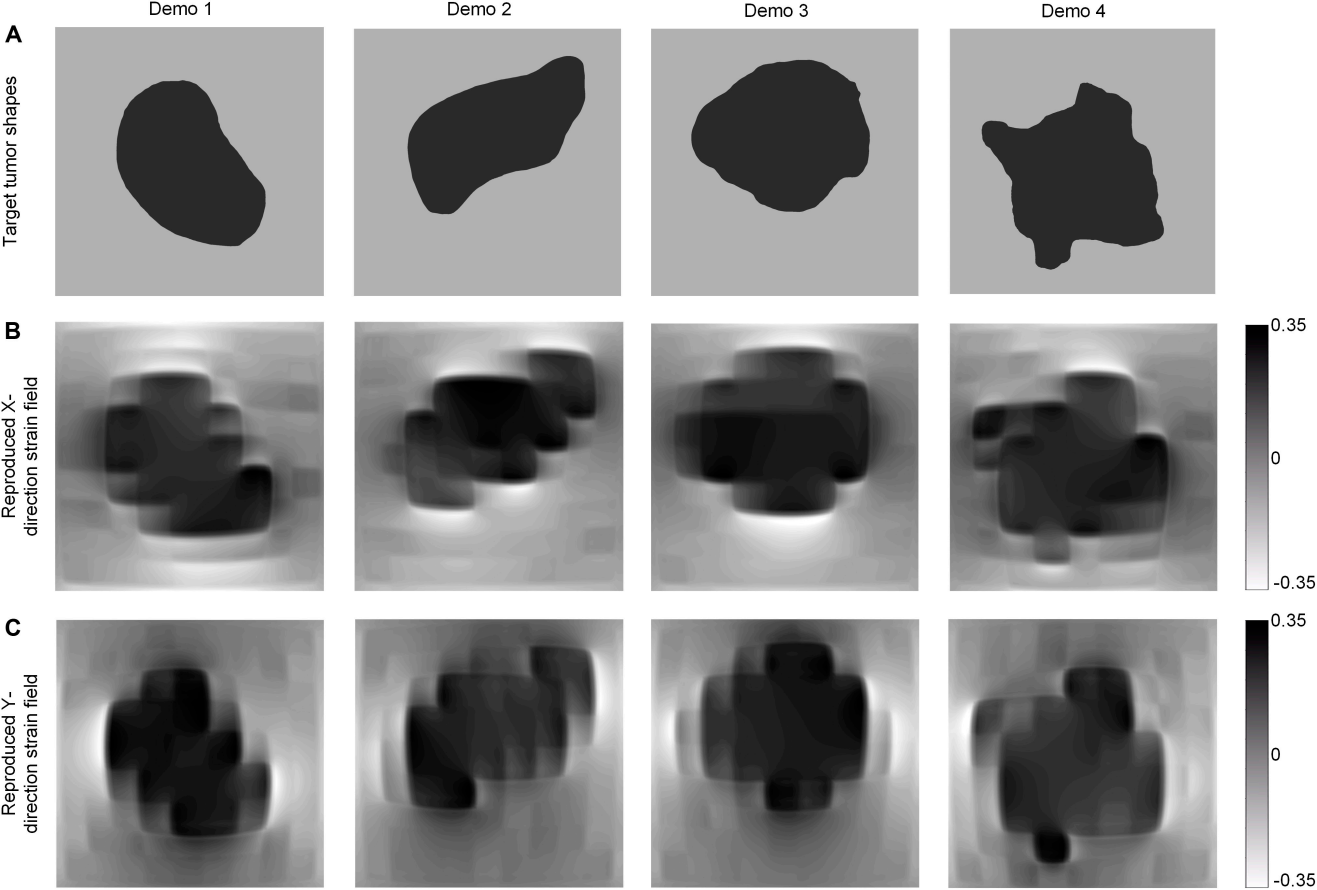
Reproducing target strain field derived from tumor–stroma interfaces for cancer cell testbed. (A) Target strain field derived from breast tumor–stroma interfaces. (B) Reproduced X-direction strain field. (C) Reproduced Y-direction strain field.

Drawing on the research by Byrd et al. [43] on breast cancer tumor–stroma interfaces, we extracted 3 different shapes of tumor–stroma interface and created target input images as shown in Fig. 7A. These were then input into the inverse control pretrained models for X-direction and Y-direction strain, respectively. The voltage input arrays obtained were applied to the virtual device, resulting in the strain replications shown in Fig. 7B and C, with Fig. 7B representing the replication of X-direction strain and Fig. 7C representing the replication of Y-direction strain. Comparing these replications with the target input images, it is evident that, despite the limitations of current resolution, our proposed method successfully replicates the input tumor–stroma interfaces. This offers a valuable testbed for biomechanical research on tumors.

We compared the similarity between the target tumor–stroma interfaces with the reproduced strain field,, and the result is shown in Table.

## Discussion

The paper successfully demonstrates the use of a 9 × 9 array of independently controllable DEAs to achieve precise control over strain fields, overcoming the limitations of traditional bioreactor technologies. Through the integration of image regression-based machine learning, both forward and inverse control methods were developed, enabling rapid prediction and replication of target strain fields. The use of FEA for data collection and the novel application of MLP and SRGAN models for machine learning-based control underscore the innovative methodologies employed in this research. Our proposed bioreactor has potentially vast applications, ranging from the study of tumor biomechanics to the exploration of cellular responses under various mechanical stimuli. By replicating biomechanically significant strain fields and customizing strain fields based on tumor–stroma interface, this bioreactor demonstrates its potential as an advanced testbed for research in mechanobiology, tissue engineering, and regenerative medicine.

This paper primarily focuses on using image-based machine learning algorithms to achieve customized control of complex strain fields, which is computationally intensive. In addition, it is important to note that unlike the study in this paper, the physical device would require insulation gaps between pixels, thus slightly modifying the coupling between pixels. The device would then be prestretched and mounted on the base after which cultured cells could be studied under modified strain fields. The FEA model corresponding to the physical model of the device would then be verified using experimental data, before using machine learning (developed on the FEA data) to try and generate the desired strain fields on the physical device. We are currently working on replicating the 6 × 6 DEA array proposed in previous studies [29] and aim to scale it up to a 9 × 9 array. Our goal is to implement our proposed control method to customize complex strain fields.

In conclusion, this paper represents a significant step forward in the customization of strain fields for biomechanical research, showcasing the potential of combining advanced materials, machine learning, and simulation techniques to address complex challenges in the field of biomechanics and beyond.

## Data Availability

All data generated or analyzed during this study are included in this published article. The source code used for the analysis is available on GitHub at the following link: https://github.com/wang5056/customizing-strain-field.
